# Network analysis on depressive symptoms and big five personality traits of community elderly over 60 years old: a cross-sectional study

**DOI:** 10.3389/fpsyt.2025.1612640

**Published:** 2025-08-21

**Authors:** Mo Zhu, Yanqun Zheng, Yuan Fang, Qi Qiu, Xia Li

**Affiliations:** ^1^ Department of Geriatric Psychiatry, Shanghai Mental Health Center, Shanghai Jiao Tong University School of Medicine, Shanghai, China; ^2^ Department of Psychiatry and Psychology, Huashan Hospital, Fudan University, Shanghai, China

**Keywords:** elderly, depressive symptoms, big five personality traits, network analysis, cross-sectional study

## Abstract

**Background:**

The global population is undergoing significant aging, with the elderly facing prominent physical and mental health challenges. Geriatric depression is becoming increasingly prevalent, imposing a heavy burden on healthcare and caregiving. This study employs network analysis to explore the relationship between geriatric depressive symptoms and the Big Five personality traits, aiming to provide a theoretical basis for preventing and intervening in geriatric depression.

**Methods:**

A total of 585 residents aged 60 and above, with an average age of 67.14 ± 5.26 years, were included in this study. The Geriatric Depression Scale-15 was used to assess depressive symptoms, and the 60-item version of the Big Five Personality Inventory was used to assess personality traits. The network model was constructed in R using the qgraph, bootnet, and networktools packages, applying LASSO regularization with EBIC for model selection. Network centrality was evaluated using Strength and Bridge Strength as indicators. Meanwhile, network comparison analyses were conducted for different genders.

**Results:**

The model included 45 edges, 29 of which had non-zero estimates, with an average edge weight of 0.038. Openness had negative connections with withdrawal apathy-vigor (WAV) and hopelessness; Conscientiousness had a negative connection with dysphoric mood; Extraversion had a negative connection with WAV; Agreeableness had a negative connection with anxiety; and Neuroticism had positive connections with dysphoric mood, WAV, anxiety, memory complaints, and hopelessness. According to the strength centrality ranking, the top four nodes were Neuroticism, Conscientiousness, dysphoric mood, and hopelessness. The nodes with higher bridge strength were Neuroticism, dysphoric mood, and WAV. The analysis stratified by gender revealed that Neuroticism consistently exhibited the highest strength and bridge strength. In terms of the strength of depressive symptoms, dysphoric mood was most prominent in males, while hopelessness was most significant in females. Regarding bridge strength, anxiety symptoms had the highest bridge strength in males, whereas dysphoric mood had the highest bridge strength in females.

**Conclusion:**

Different personality traits show varied associations with geriatric depressive symptoms. Neuroticism is crucial in the Personality–Depressive Symptoms network, and gender differences exist in this relationship. These findings may offer guidance for the prevention and treatment of depressive symptoms in older adults.

## Introduction

In recent years, the accelerating global aging population has led to significant demographic shifts, posing new challenges to society development (1). These changes have also introduced numerous difficulties in the lives of the elderly, with their physical and mental health issues becoming increasingly prominent (2). Among these issues, depression has emerged as a major mental health concern for the elderly, making it a critical issue in the field of geriatric health. Depression is highly prevalent among the elderly, with a global prevalence of 30.2-40.4% (3). Data indicate that the global number of depression has risen from 172 million in 1990 to 258 million in 2017, representing a 49.86% increase (4). Furthermore, the growing number of people with depression places a significant economic and social burden on society. It is predicted that by 2030, depression will become the leading cause of the global disease burden, with approximately 273.8 million people suffering from depression worldwide (5). It is worth noting that there are more elderly individuals who exhibit symptoms of depression but do not meet the criteria for a depressive disorder. In China, 20.3% of the elderly meet the diagnostic criteria for depressive disorders, and 30.6% exhibit significant depressive symptoms (6, 7). Given that the elderly are generally less likely to express their discomfort, these symptoms can be easily overlooked to some extent. This highlights the need for us to pay close attention to their emotional issues. Therefore, exploring issues related to geriatric depression symptoms and identifying effective coping strategies have become crucial public health tasks.

During the geriatric phase, the emergence of depressive symptoms is not only related to physical decline and changes in social relationships, but is more closely associated with psychological factors (8, 9). As a core psychological characteristic in an individual’s cognitive, emotional, and behavioral patterns, personality traits are particularly significantly associated with depressive symptoms. Although personality traits are relatively stable over a given period, they are not immutable across the entire lifespan (10). In elderly, personality traits exhibit unique distribution patterns and manifestations that distinguish them from those of other age groups (11–13). For instance, unique experiences and changes during the geriatric phase can lead to alterations in everyday thoughts, emotions, and behaviors, which can, over time, result in changes in personality traits.

Previous research has revealed that the Big-Five Personality model (14), a key indicator of individual personality traits, encompasses five dimensions: Neuroticism, Extraversion, Openness, Agreeableness, and Conscientiousness, all of which are closely associated with emotional symptoms (15, 16). However, these five personality traits each play different and inconsistent roles in depression. High levels of Agreeableness and Openness have been found to reduce psychological distress and sadness, while Conscientiousness has the opposite effect (17). Koorevaar et al. (18) found that Neuroticism is a risk factor for late-life depression, and Extraversion and Conscientiousness are protective factors against it, and notably, there is an association between high Openness and earlier age of depression onset. Lyon et al. (19) found that the depression facet of Neuroticism, the positive emotion and assertiveness facets of Extraversion, and the competence facet of Conscientiousness explain the variance in anxiety and depressive symptoms in a community sample. Moreover, the correlations differ among different age groups. For example, the research found that the negative correlations between Conscientiousness, Agreeableness and depressive symptoms are the strongest in the elderly group, and gradually weaken in the middle-aged group and the young group in sequence (20). Moreover, personality traits also show a significant correlation with the severity of depressive symptoms in the elderly (21). It is important to note that the interrelationships and combined effects among different personality traits can impact depressive symptoms. For instance, the “worst two out of three” principle suggests that the presence of two high-risk personality traits can offset the protective effect of a low-risk trait (22). Over all, the relationship between personality traits and depressive symptoms is intricate and highly significant. Given that the unique characteristics of depressive symptoms in older adults (23), it is essential to adopt a systemic perspective to dissect the underlying connections between personality traits and depressive symptoms, thereby identifying core symptoms that can inform targeted interventions.

Network analysis can effectively reveal the complex relationships between variables. The psychosocial network model conceptualizes mental health problems as a complex system of interrelated symptoms, providing an alternative analytical framework for relevant research (24, 25). In network analysis, variables are represented as nodes, with their interrelationships depicted as edges (26). This framework empowers researchers to delve into the structure and connectivity of complex systems. It also reveals the importance of nodes and identify the central symptoms through centrality calculation (27). Bian et al. (28) found that among the Chinese elderly, “nervousness” and “sad mood” exhibit high strength centrality, emerging as the primary indicators of emotional symptoms. Additionally, Shang et al. (29) found that intervening on “emotional representation” as a core symptom could potentially alleviate psychological problems in elderly individuals within community populations. In the field of mental health research, network analysis can be used to deeply analyze the interactions between psychological symptoms, personality traits, and social environmental factors (30). Although there has been a wealth of research on the relationship between depressive symptoms and the Big Five personality traits (31, 32), studies using network analysis methods in elderly person are relatively scarce. Specifically, network analyses exploring the links between depressive symptoms and the Big Five traits in older adults remain notably scarce. Therefore, this study aims to use network analysis methods to explore the relationship between depressive symptoms and the Big Five personality traits in the elderly, aiming to provide a robust theoretical foundation for the prevention and intervention of geriatric depression.

## Methods

### Participants

This study retrospectively analyzed residents aged 60 years and older who participated in the health and cognitive screening program in Yinhang Community, Shanghai, in 2017. Trained assessors collected comprehensive demographic data from the community residents and evaluated their neuropsychological status such as depressive mood, and personality traits. A total of 585 participants who completed the assessments were included in this study.

### Inclusion criteria

Participants were required to be aged 60 years or above, regardless of gender. They had to have no severe physical ailments, normal eyesight and hearing, and the ability to read, write, and communicate effectively in language, as well as the capacity to complete questionnaire assessments and screenings. Additionally, they must have consented to provide relevant clinical data.

### Research instruments

#### Depression assessment

The 15-item Geriatric Depression Scale (GDS-15) was used to assess the emotional state (33, 34). This scale consists of 15 items, with responses scored as “yes” or “no”. Each item is assigned 0 or 1 point (with items 1,5,7,11, and 13 scored in reverse), and the total score ranges from 0 to 15. Higher scores indicate more severe depressive symptoms (35, 36). Although different items represent distinct symptom manifestations, individual symptom items alone are insufficient to capture the commonalities between symptoms. Previous research has addressed this limitation by employing dimensionality reduction techniques to categorize the GDS-15 into five distinct dimensions, which more effectively represent the overall symptom profile. In this study, GDS-15 was further divided into five subdimensions, namely dysphoric mood, withdrawal-apathy-vigor (WAV), anxiety, memory complaints, and hopelessness (37). In this study, the Cronbach’s α coefficient for the GDS-15 was 0.77, indicating good reliability.

#### Personality trait assessment

The 60-item Big Five Personality Inventory was used to assess personality traits (14). Based on the Big Five personality theory, this inventory covers five major personality dimensions. Openness reveals how receptive individuals are to new ideas, experiences, and creative expressions. Conscientiousness highlights a person’s self - discipline, organization, and strong sense of duty, often leading them to be highly reliable in fulfilling tasks and commitments. Extraversion showcases an individual’s sociability, their preference for being around others, and their ability to draw energy from social interactions, making them the life of social gatherings. Agreeableness reflects a person’s kindness, empathy, and willingness to cooperate, enabling them to build harmonious relationships by considering others’ feelings. Neuroticism indicates the degree of emotional instability, with those high in this trait being more likely to experience frequent mood swings and heightened sensitivity to stress. The scale consists of 60 items, with 12 items corresponding to each personality dimension. Each item is scored on a scale from 0 to 4, with higher scores indicating a more pronounced expression of the respective personality trait in the elderly (14, 38). In this study, the Cronbach’s α coefficient for the inventory was 0.86, indicating good reliability.

### Statistical analysis

Data analysis was performed using SPSS version 24.0 for descriptive statistics. Continuous variables were described using mean ± standard deviation (x ± s), while categorical variables were presented as percentages. Network estimation was conducted using the “qgraph”, “bootnet”, and “networktools” packages in R version 4.4.2.

Initially, the network model was constructed using the LASSO regularization method, with the Extended Bayesian Information Criterion (EBIC) employed to select the optimal model. In this network model, GDS symptoms and personality traits were defined as nodes, while edges represented the associations between two variables after controlling for the influence of other variables in the network. The thickness of the edges intuitively reflected the strength of the associations between symptoms, with thicker edges indicated stronger weights. To ensure the accuracy and validity of the network construction, the tuning parameter was set at 0.5. For network centrality assessment, node strength was selected as the key indicator, defined as the sum of the absolute weights of all edges directly connected to a given node. A higher node strength value indicated a closer direct association with neighboring nodes, suggesting a more central and critical position within the network structure (39). For assessing the centrality of bridge symptoms, bridge strength was used. This metric specifically reflected the total direct connections of bridge symptoms with other symptom clusters, with a higher value indicated a greater role in connecting different symptom clusters (40). Finally, to ensure the stability and reliability of the constructed network, edge weight estimation was performed by bootstrap method with 1000 bootstraps, and the Correlation Stability coefficient (CS-c) was calculated using a bootstrap procedure with 2500 bootstraps (41).

## Results

### General demographic data

A total of 585 community-dwelling elderly individuals were included in this study, with a mean age of 67.14 ± 5.26 years. Among them, 227 were male (38.8%) and 358 were female (61.2%). Regarding personality traits, the mean scores were as follows: Openness, 26.16 ± 4.52; Conscientiousness, 31.94 ± 5.03; Extraversion, 28.42 ± 5.81; Agreeableness, 31.84 ± 4.07; and Neuroticism, 17.31 ± 5.79. The mean GDS-15 score was 3.13 ± 2.87. Detailed information is shown in **Table 1**.

**Table 1:** General demographic information.

N	Age	Gender [N(%)]		Openness	Conscientiousness		Extraversion	Agreeableness	Neuroticism		GDS-15
		Male	Female								
585	67.14 ± 5.26	227 (38.8)	358 (61.2)	26.16 ± 4.52	31.94 ± 5.03		28.42 ± 5.81	31.84 ± 4.07	17.31 ± 5.79		3.13 ± 2.87

*GDS-15*. The 15-item Geriatric Depression Scale.

### Network analysis and model construction

**Figure 1:**
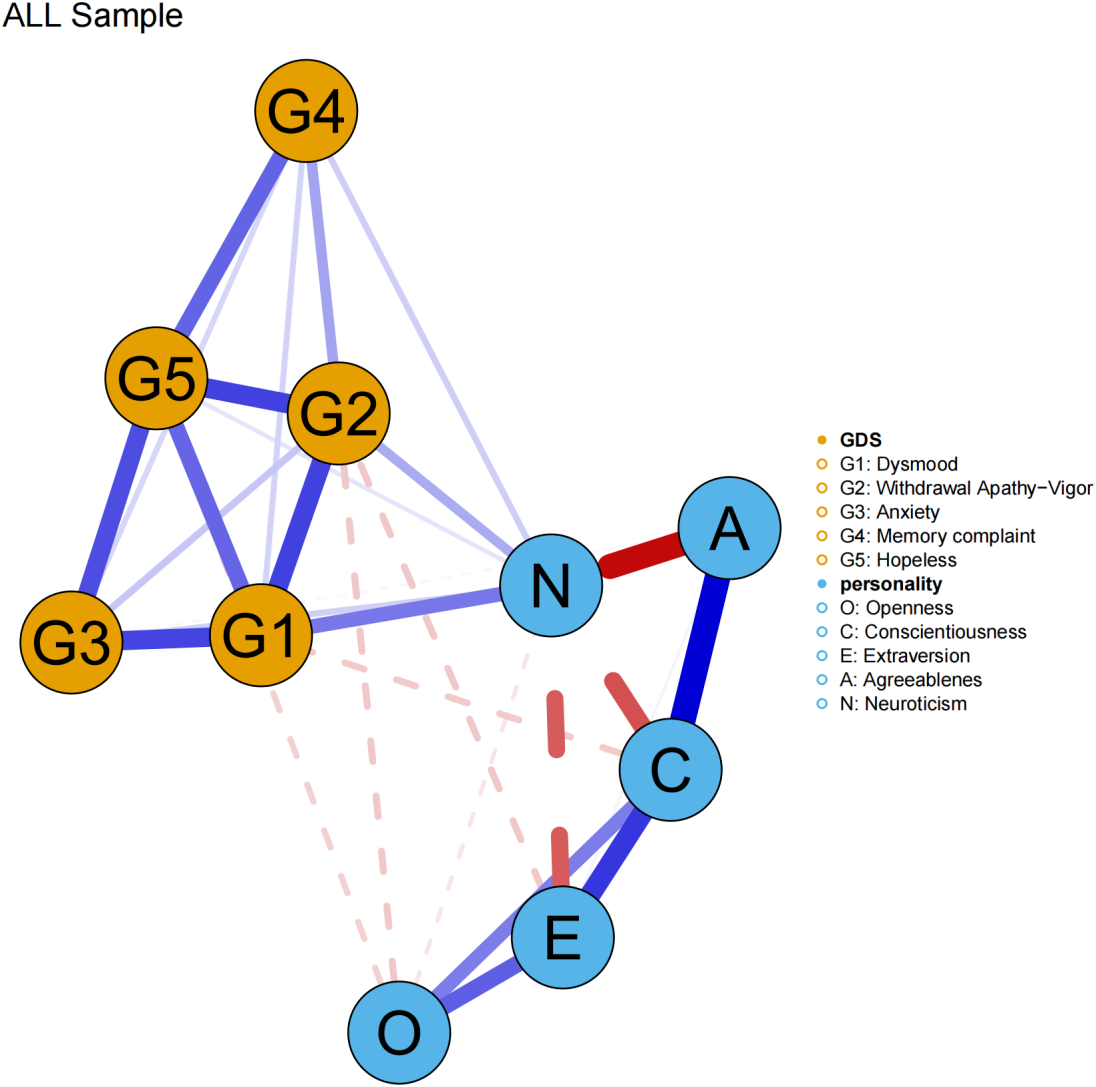
The network structure of Big Five personality traits and depressive symptoms. The thicker the node line, the stronger the connection between symptoms; the blue solid line indicates a positive connection, and the red dashed line indicates a negative connection.

Using network analysis, we constructed a network model linking the Big Five personality traits and depressive symptoms (hereafter referred to as the Personality-Depression network), which was depicted in **Figure 1**. The analysis revealed that out of a total of 45 possible edges, 29 edges had non-zero estimates, with an average edge weight of 0.038. Among the two clusters of personality traits and depressive symptoms, Openness was negatively connected with two depressive dimensions. Specifically, the edge weight between Openness and WAV was the highest at -0.06, followed by the edge weight between Openness and hopelessness at -0.05. Conscientiousness was negatively connected to dysphoric mood, with an edge weight of -0.058. Extraversion was negatively connected to WAV, with an edge weight of -0.06. Agreeableness was negatively connected to anxiety, with an edge weight of -0.01. Neuroticism exhibited positive connections with all five dimensions of depressive symptoms. The strongest connection was between Neuroticism and dysphoric mood, with an edge weight of 0.148, followed by a connection to WAV with an edge weight of 0.092. Detailed connections and edge weights are presented in **Table 2**.

**Table 2:** Edge weights of the personality – depression symptoms network structure.

Variables	Openness	Conscientiousness	Extraversion	Agreeableness	Neuroticism	G1	G2	G3	G4	G5
Openness	–									
Conscientiousness	0.142	–								
Extraversion	0.176	0.219	–							
Agreeableness	0	0.278	0.012	–						
Neuroticism	-0.028	-0.191	-0.178	-0.269	–					
G1	0	-0.058	0	0	0.148	–				
G2	-0.06	0	-0.06	0	0.092	0.207	–			
G3	0	0	0	-0.01	0.051	0.203	0.062	–		
G4	0	0	0	0	0.053	0.048	0.101	0.045	–	
G5	-0.05	0	0	0	0.03	0.168	0.207	0.195	0.17	–

*G1*: dysphoric mood; *G2*: withdrawal apathy-vigor; *G3*: anxiety; *G4*: memory complaints; *G5*: hopelessness.

### Centrality result

**Figure 2:**
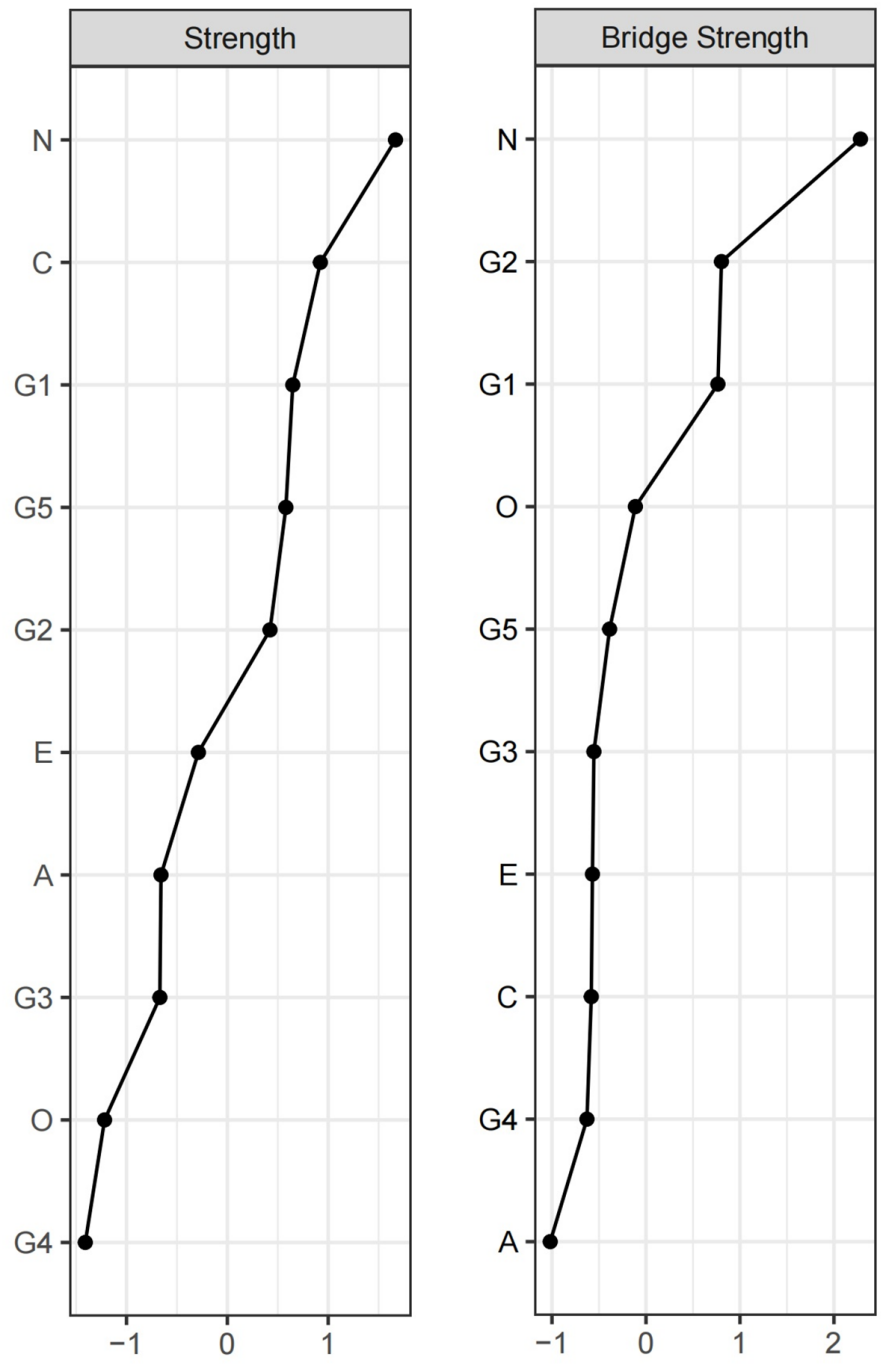
Strength and Bridge Strength of Each Node in the Network. O, Openness; C, Conscientiousness; E, Extraversion; A, Agreeableness; N, Neuroticism; G1, dysphoric mood; G2, WAV; G3, anxiety; G4, memory complaints; G5, hopelessness.

After calculating the centrality indices for each node, the top four nodes ranked by strength centrality were Neuroticism, Conscientiousness, dysphoric mood and hopelessness. Neuroticism and Conscientiousness were personality dimensions, while dysphoric mood and hopelessness were depressive symptom dimensions. This indicates that these nodes exerted significant influence in the network through their connections with other nodes. The nodes with the highest bridge strength were Neuroticism, dysphoric mood, and WAV. These results suggest that, within the Personality-Depression network structure, Neuroticism plays a prominent role in bridging the personality and depressive symptom clusters. Among depressive symptoms, dysphoric mood and WAV exhibit strong bridging functions between the two clusters. See **Figure 2** for details.

### Stability results

**Figure 3:**
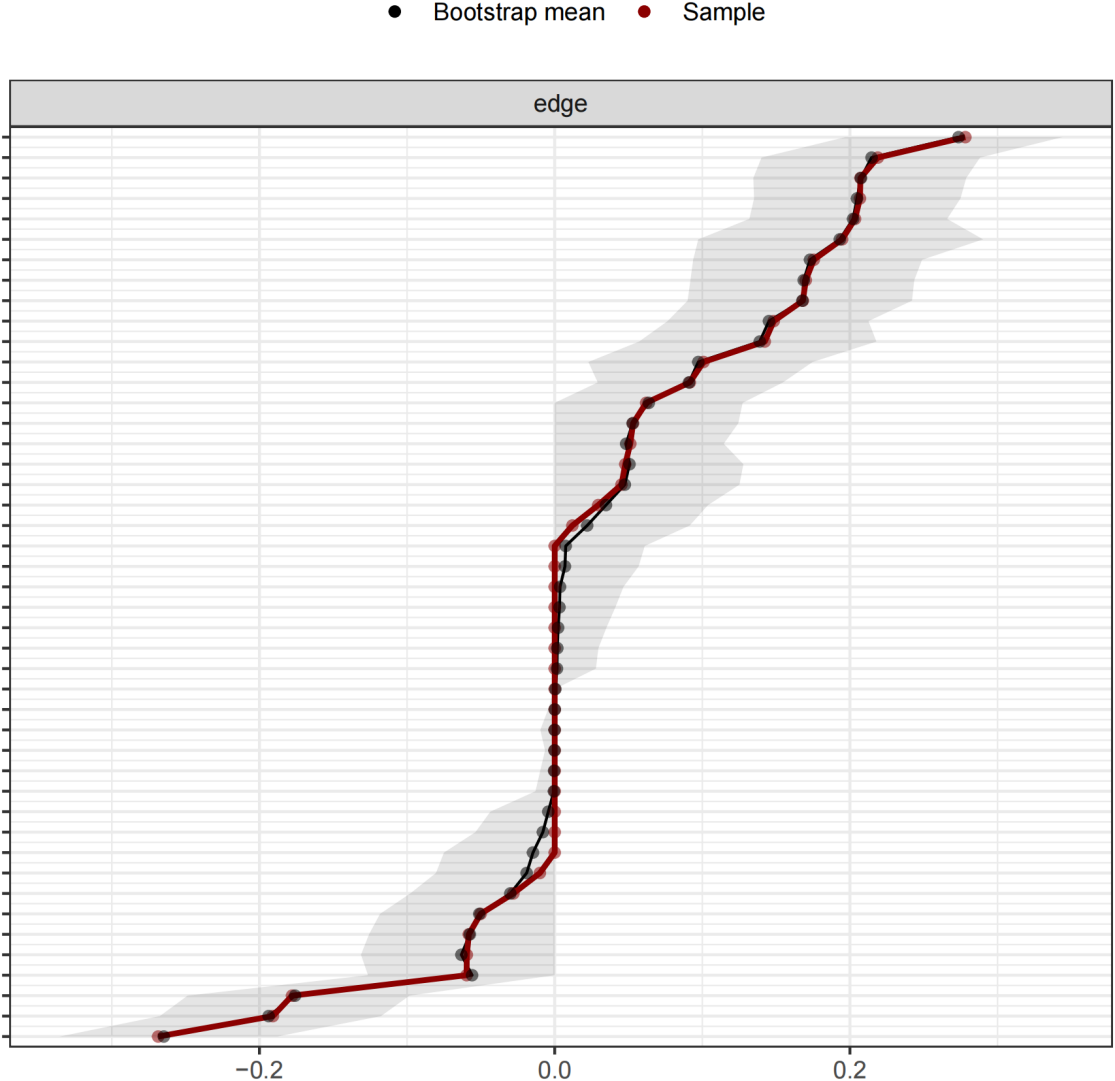
Bootstrapped confidence intervals of edge weights. Red represents the connection weight of the symptom network in this study. Black represents the mean value of the edge weights calculated by Bootstrap. The gray area represents the 95% confidence interval (CI).

**Figure 4:**
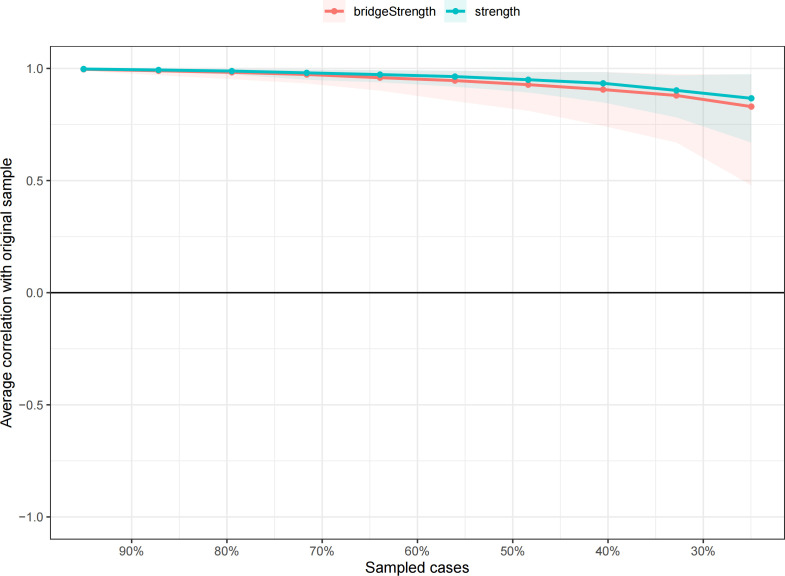
The stability of strength and bridge strength using case-dropping bootstrap.

The edge weights calculated using the Bootstrap method were found to overlap closely with the sample weights, and the 95% confidence intervals were relatively narrow. This indicates that the network model is highly reliable and that the estimation of edge weights is accurate (**Figure 3**). The Correlation Stability coefficient (CS-c) for node strength was 0.75, and the correlation coefficient for bridge strength was 0.672, both of which exceeded 0.5. These results demonstrate that the network structure constructed in this study is highly stable (**Figure 4**).

### Gender differences in network structure

**Figure 5:**
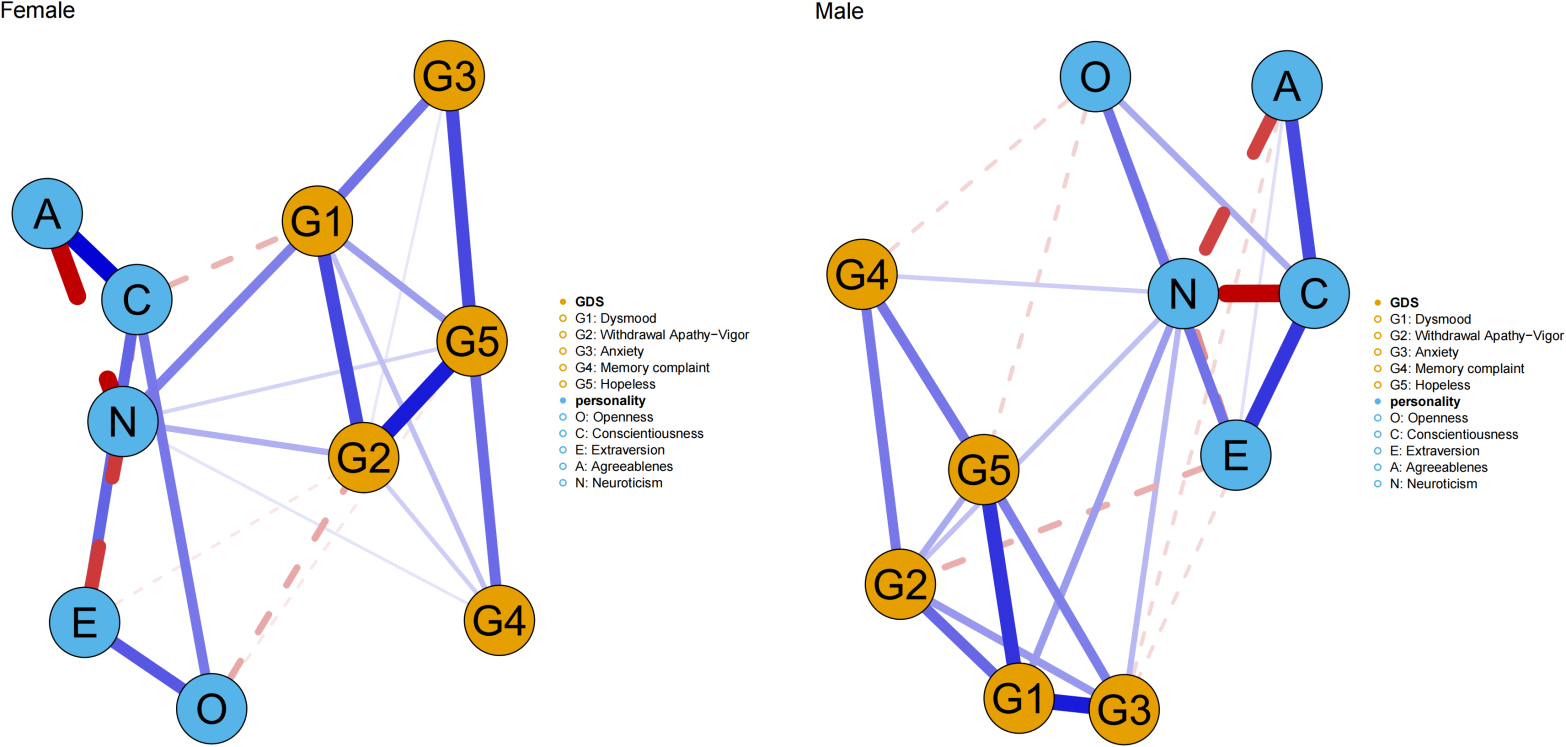
The network structure of Big Five personality traits and depressive symptoms between male and female. The thicker the node line, the stronger the connection between symptoms. The blue solid line indicates a positive connection, and the red dashed line indicates a negative connection.

Further analysis of the personality-depression network structure revealed differences between males and females. In the network of male, the distribution was largely similar to that of the overall population. Out of the total 45 edges, 27 had non-zero estimates, with an average edge weight of 0.034. Specifically, Openness was negatively correlated with subjective memory decline but had no connection with WAV. Conscientiousness was connected with anxiety symptoms but not with dysphoric mood. Neuroticism was not connected with hopelessness. In the network of female, 26 out of 45 edges had non-zero estimates, with an average edge weight of 0.036. The only notable difference was the absence of a connection between Agreeableness and anxiety symptoms in females, while other aspects remained similar to the overall population distribution. See **Figures 5** for details.

**Figure 6:**
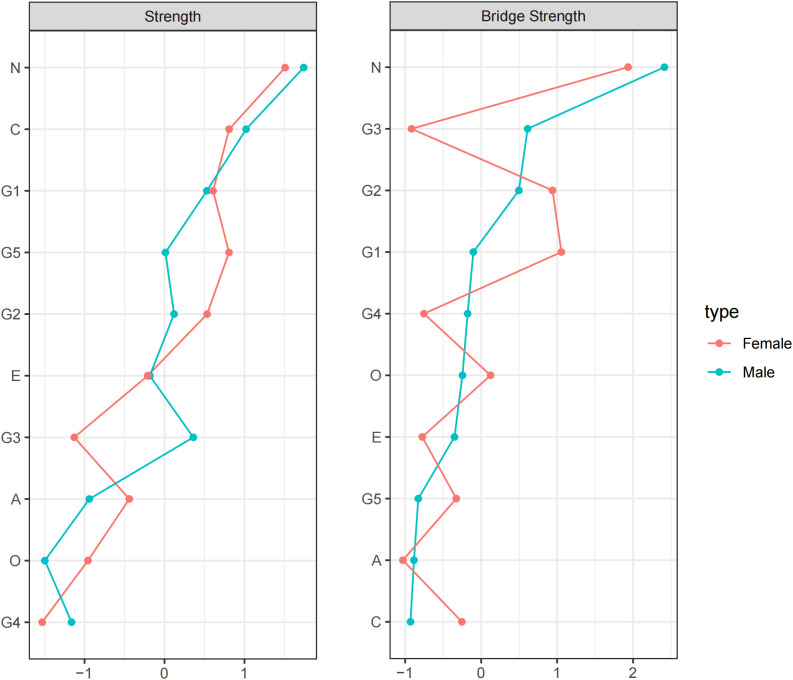
Strength and Bridge Strength of Each Node in the Network between male and female. O, Openness; C, Conscientiousness; E, Extraversion; A, Agreeableness; N, Neuroticism; G1, dysphoric mood; G2, WAV; G3, anxiety; G4, memory complaints; G5, hopelessness.

In terms of centrality analysis by gender, Neuroticism and Conscientiousness consistently ranked highest among personality traits in terms of strength. Among depressive symptoms, dysphoric mood had the highest strength in males, followed by anxiety and WAV. In females, hopelessness had the highest strength, followed by dysphoric mood and WAV. In bridge strength, Neuroticism emerged as the personality trait with the highest bridge strength in both genders. For males, anxiety and WAV were the depressive symptoms with the highest bridge strength, while for females, dysphoric mood and WAV were the most prominent. See **Figures 6** for details.

## Discussion

This study aimed to explore the network associations between the Big Five personality traits and depressive symptoms in the elderly and successfully constructed a Big Five Personality-Depressive symptoms network model. The findings revealed that among the 45 edges in the network model, 29 were non-zero edges, indicating multiple association patterns between different personality traits and depressive symptoms. For example, Openness was negatively connected with certain depressive dimensions, while Neuroticism was positively connected with multiple depressive dimensions. Centrality and bridge analyses identified key nodes within the Personality-Depression network. Among personality traits, Neuroticism emerged as the most central connecting node, while among depressive symptoms, dysphoric mood and WAV were the two primary connecting nodes. The network model demonstrated high reliability and stability.

In the field of research exploring the relationship between personality traits and depressive symptoms, numerous studies have demonstrated a significant positive correlation between Neuroticism and depressive symptoms. Individuals with higher levels of Neuroticism are more likely to experience depression, and high Neuroticism is associated with various depressive symptoms, such as anhedonia and anxiety (42). This phenomenon may be attributed to the fact that individuals with higher levels of Neuroticism tend to be more sensitive to emotional experiences and have less stable emotional regulation. As Neuroticism increases, individuals with varying levels of emotional awareness are likely to experience heightened negative emotions. Notably, when both Neuroticism and emotional awareness are elevated, the degree of anxiety and stress exhibited by these individuals is most pronounced (43). Research has shown that individuals with high Neuroticism have a significantly increased risk of developing depression, with a hazard ratio (HR) as high as 6.13, ranking among the top conditions associated with high Neuroticism (44). Another study revealed that age, cognitive function, social inhibition, and Neuroticism are all significantly associated with depression, with high Neuroticism being the strongest predictor of depression risk (45). These findings align with our study, where Neuroticism was positively connected to all five dimensions of depressive symptoms. Within the Personality-Depression network, Neuroticism not only had high strength in the overall network but also played the most significant role in connecting personality traits with depressive symptoms. High Neuroticism is characterized by heightened emotional reactivity and relatively weaker emotional regulation capabilities. This trait enhances sensitivity to stress and impairs the regulation of negative emotions, which directly leads to increased perceived stress (46). Research has shown that in older adults, elevated perceived stress and declining cognitive function may mediate the effect of Neuroticism on depressive symptoms (47). Concurrently, elderly individuals with higher levels of Neuroticism are more prone to experiencing negative emotions such as anxiety and anger. Trofimova (48) found that Neuroticism impacts emotions via imbalanced opioid receptor regulation, with its exacerbation potentially causing emotional problems. Joseph et al. (49) further revealed that compared with non-neurotic depressed elders and non-depressed controls, high-Neuroticism depressed elders had reduced volumes in frontal lobe regions (e.g., frontopolar, medial orbitofrontal cortex, left orbital gyrus), indicating the critical role of frontal brain areas in Neuroticism-related depressive symptoms. This trait is not confined to the realm of psychology, as it also has significant associations in the field of genetics. Adams et al. (50) analyzed genome-wide association study (GWAS) data and identified overlapping genomic regions for depression and Neuroticism, further clarifying their specific genetic correlations with other traits after statistical adjustment. Similarly, Tubbs et al. (51) showed that parental genetic profiles not only directly influence offspring depression risk but also elevate this risk indirectly by shaping offspring Neuroticism through family environmental mediation. Moreover, Ye et al. (52) used Mendelian randomization to confirm a causal link between Neuroticism and aging phenotypes, which are closely tied to emotional symptoms through impaired cognition, induced hopelessness, and reduced vitality—thereby increasing depressive vulnerability.

In our study, Openness was found to be negatively associated with reduced vitality and feelings of hopelessness. This finding aligns with prior research, which has shown no clear correlation between general dysphoric mood and Openness, while specific sub-dimensions of Openness are linked to depressive symptoms (53). Additionally, Heisel et al. (54) found that Openness moderates the relationship between hopelessness and suicidal ideation. Low Openness can diminish patients’ willingness to report suicidal thoughts, potentially lowering clinicians’ alertness and increasing suicide risk.

Conscientiousness was found negatively correlated with dysphoric mood. This is consistent with a previous study involving 2,318 older adults from the English Longitudinal Study of Ageing (55). Conscientiousness is highly related to culture background. In traditional Chinese culture, Confucianism emphasize self-restraint and ethical conduct, which may foster Conscientiousness. High Conscientiousness, in turn, is associated with more regular routines and habits, both of which can serve as protective factors against depression. Furthermore, we found that Agreeableness was negatively correlated with anxiety symptoms. This aligns with previous findings that, after controlling for depressive symptoms and other sociodemographic covariates, anxiety is positively correlated with Neuroticism and negatively correlated with Agreeableness and Conscientiousness (56). Individuals with high Agreeableness are more likely to establish close relationships and actively seek support from others. Social support, in turn, serves as a significant buffer against anxiety (57). Our study also revealed a negative correlation between Extraversion and reduced vitality, a finding that has been reported in related studies (58). Individuals with high Extraversion typically possess more energy and engage in more activities, which may help them maintain vitality.

However, our findings also differ from those of previous studies. This discrepancy may be attributed to the fact that our study focused on the elderly population, whereas many previous studies have primarily involved younger adults. Older adults possess unique physiological and psychological characteristics, such as progressive physical decline, psychological and cognitive changes resulting from life experiences, and shifts in social roles (11, 59). These factors may lead to distinct relationships between personality traits and depression in older adults compared to other age groups. It is important to note that the impact of personality traits on depression is not solely direct. Social networks, as represented by the number of friends and relatives, play a mediating role in this relationship (60). This suggests that personality traits do not influence depressive symptoms in isolation but rather interact with social networks to collectively affect an individual’s mental health status.

In terms of gender differences, distinct patterns are observed in the relationship between personality traits and depression. Prenatal androgen exposure during mid-gestation induces permanent functional and structural changes in male brain, shaping masculine personality traits (61). This may serve as the biological basis for the observed differences between genders. Moreover, the differences in social roles and social identity also have varying impacts on personality shaping. In this study, we found that the connection between Neuroticism and dysphoric mood was the strongest among females, while the connection between Neuroticism and anxiety was more pronounced among males. Neuroticism has been confirmed as a marker of emotional instability, and individuals with high Neuroticism, regardless of gender, tend to experience more unstable emotions. The prominent distinction between genders may be due to the differences in social expectations and psychological resilience (62, 63). In traditional culture, older women are often ascribed the role of caregivers. The negative association between dysphoric mood and Conscientiousness observed in this study may stem from such social expectations and role conditioning, which not only promote heightened Conscientiousness but also entail elevated self-demands and the effort required to meet these standards. Among the personality traits directly connected to anxiety in men, Extraversion and Agreeableness show negative correlations with anxiety, in addition to Neuroticism. This finding is consistent with previous research. Although men are traditionally expected to take on the role of independent problem-solvers, more extraverted individuals can break through this stereotype by seeking more support and thereby alleviating their stress. Agreeableness, on the other hand, represents cooperation and trust, which means that individuals with higher Agreeableness are less likely to suppress themselves. These differences can influence the relationship between personality traits and depression.

Our study found hopelessness strongly influenced network structure in elderly women. Generally, women are more inclined to express their emotions and seek social support in relationships. When facing psychological stress, they are more likely to alleviate their emotions by sharing their feelings and experiences with others. However, when women are in a state of depression, their emotional state and negative thinking can hinder their willingness and ability to seek help, cutting off potential avenues for emotional relief and potentially exacerbating their feelings of hopelessness (64). In contrast, among elderly men, anxiety exerted a more pronounced influence on the network, and link the Personality-Depression networks. This may be attributed to the fact that men may be more accustomed to internalizing stress and attempting to deal with problems independently (65). However, when confronted with late-life challenges, men are less inclined to proactively seek external support, a tendency influenced by traditional masculine norms that emphasize self-reliance and emotional stoicism (66). This behavioral pattern may exacerbate mental health issues by perpetuating internalized stress and impeding the timely intervention and management of psychological distress (67).

This study has several limitations. While the selected community boasts a large population with heterogeneous demographic characteristics, its single-community design may compromise generalizability. Future research could adopt multi-community sampling to enhance representativeness. Additionally, the cross-sectional study hinders rigorous causal inference. To address this, we intend to conduct longitudinal follow-up studies building on current findings, aiming to clarify the dynamic interplay between personality traits and depressive symptoms. Moreover, although the GDS-15 used here is a validated self-report measure, its reliance on subjective assessment introduces potential bias. Future studies should integrate objective indicators via multi-omics approaches to mitigate this limitation. Furthermore, the lack of multiple testing corrections may have inflated associations or introduced false positives. Finally, expanding the sample size and incorporating additional confounding factors will further refine the accuracy and generalizability of these findings.

## Conclusion

This study successfully constructed a network model linking the Big Five personality traits to depressive symptoms in community-dwelling elderly individuals. The findings revealed that Neuroticism serves as a key connecting node within this network, playing a crucial role in bridging personality traits and depressive symptoms. Additionally, significant differences in node strength and bridge strength were observed between genders. These results provide valuable theoretical support for understanding the pathogenesis of geriatric depression, facilitating the development of early screening tools and personalized intervention programs targeting geriatric depression. Ultimately, this work aims to improve the mental health of older adults and reduce the incidence of depression.

## Data Availability

The raw data supporting the conclusions of this article will be made available by the authors, without undue reservation.
